# Supplementary material for: Controllable Preparation of Superparamagnetic Fe3O4@La(OH)3 Inorganic Polymer for Rapid Adsorption and Separation of Phosphate
Source: Polymers (Basel). 2023 Jan 3;15(1):248. doi: 10.3390/polym15010248 (PMC9824844; doi:10.3390/polym15010248)
Supplement: Supplementary file 1 [file polymers-15-00248-s001.zip › polymers-2105814-supplementary.pdf]

Supplementary Information: Controllable Preparation of Superparamagnetic Fe_3O_4@La(OH)$_3$ Inorganic Polymer for Rapid Adsorption and Separation of Phosphate

Yao Lu [1], Xuna Jin [1], Xiang Li [2], Minpeng Liu [1], Baolei Liu [2], Xiaodan Zeng [3], Jie Chen [3], Zhigang Liu [3,*], Shihua Yu [1,*] and Yucheng Xu [4]

[1] Jilin Institute of Chemical Technology, College of Chemical & Pharmaceutical Engineering, Jilin 132022, China
[2] Jilin Institute of Chemical Technology, School of Petrochemical Technology, Jilin 132022, China
[3] Jilin Institute of Chemical Technology, Centre of Analysis and Measurement, Jilin 132022, China
[4] Railway Transportation Department, Jilin Petrochemical Company, Jilin 132021, China
* Correspondence: lzg@jlict.edu.cn (Z.L.); ysh@jlict.edu.cn (S.Y.)

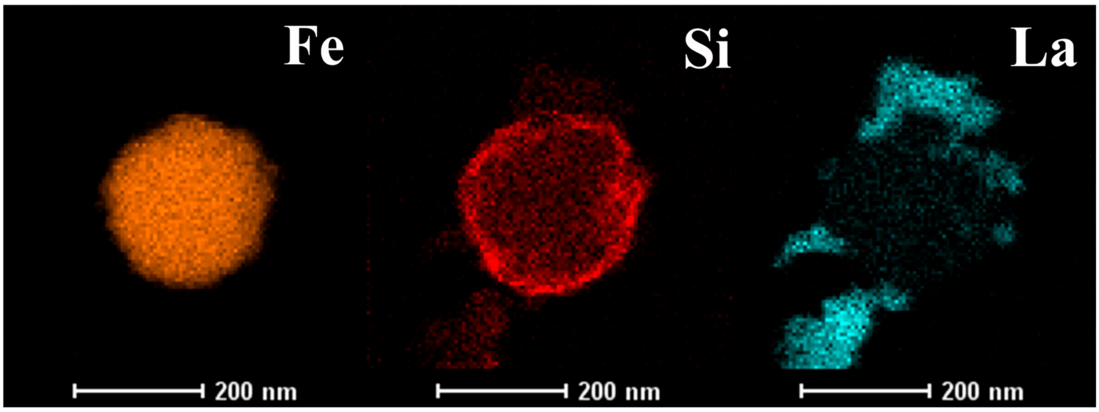

Figure S1 EDX elemental mapping of Fe_3O_4@La(OH)$_3$.

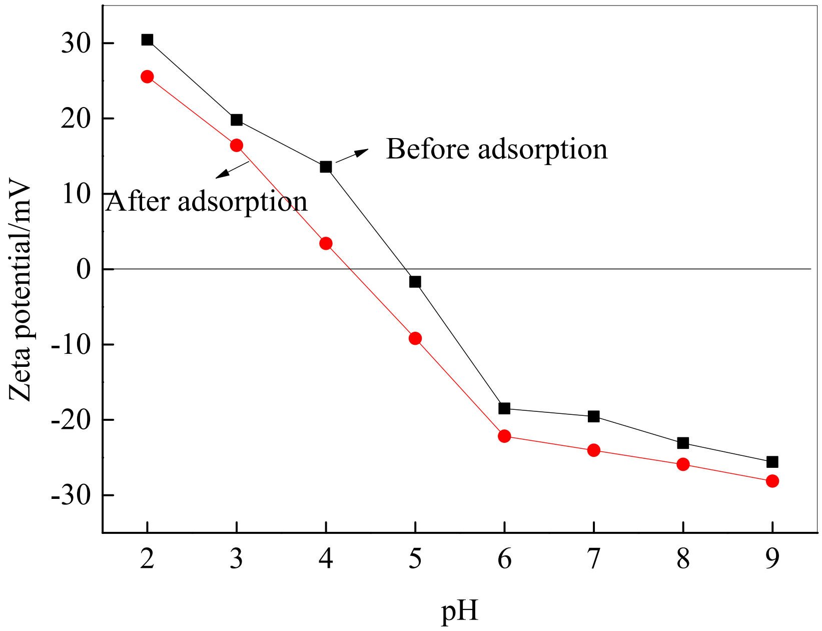

Figure S2 Zeta potential curves of Fe_3O_4@La(OH)$_3$ nanoparticles before (1) and after (2) the phosphate adsorption with the initial phosphate concentration of 30 mg/l.

Table S1 Comparison of phosphate adsorption capacities with other reported adsorbents.

Adsorbent	equilibration time	Adsorption capacity	Removal rate	reference
Calcium silicate hydrate	120 min	65.42 mgPO_4^{3-}/gC-S-H)	>98%	Journal of Environmental Management 301 (2022) 113923.
alginate-like exopolymers	240 min	1.22±0.07 mg PO_4^{3-}-P/gTS_{ALE}	90.8%	Bioresource Technology 333 (2021) 125167.
Fe_3O_4/Mg_2Al-NO_3 -LDH	2 h	33.4 mgP/g	58%(1.0 g/L)	Journal of Environmental Chemical Engineering 4 (2016) 984-991.
Fe_3O_4@ZrO_2	25 min	35.0 mgP/g	___	Chemical Engineering Research and Design 145 (2019) 194–202.
CS-Li@Fe_3O_4	50 min	95.5 mgP/g (200 mg/L)	98%, at low phosphate concentrat -ion	Biochemical Engineering Journal 187 (2022) 108662.
Fe_3O_4@La$(OH)_3$	20 min	63.72 mg P/g (30 mg/L)	＞95.7%	This work
